# Lessons learned: overcoming common challenges in reconstructing the SARS-CoV-2 genome from short-read sequencing data via CoVpipe2

**DOI:** 10.12688/f1000research.136683.1

**Published:** 2023-09-01

**Authors:** Marie Lataretu, Oliver Drechsel, René Kmiecinski, Kathrin Trappe, Martin Hölzer, Stephan Fuchs

**Affiliations:** 1 Genome Competence Center (MF1), Robert Koch Institute, Berlin, 13353, Germany

**Keywords:** SARS-CoV-2, genome reconstruction, whole-genome sequencing, short reads, Illumina, amplicons, WGS, Nextflow pipeline, virus bioinformatics

## Abstract

**Background:** Accurate genome sequences form the basis for genomic surveillance programs, the added value of which was impressively demonstrated during the COVID-19 pandemic by tracing transmission chains, discovering new viral lineages and mutations, and assessing them for infectiousness and resistance to available treatments. Amplicon strategies employing Illumina sequencing have become widely established for variant detection and reference-based reconstruction of SARS-CoV-2 genomes, and are routine bioinformatics tasks. Yet, specific challenges arise when analyzing amplicon data, for example, when crucial and even lineage-determining mutations occur near primer sites.

**Methods:** We present CoVpipe2, a bioinformatics workflow developed at the Public Health Institute of Germany to reconstruct SARS-CoV-2 genomes based on short-read sequencing data accurately. The decisive factor here is the reliable, accurate, and rapid reconstruction of genomes, considering the specifics of the used sequencing protocol. Besides fundamental tasks like quality control, mapping, variant calling, and consensus generation, we also implemented additional features to ease the detection of mixed samples and recombinants.

**Results:** We highlight common pitfalls in primer clipping, detecting heterozygote variants, and dealing with low-coverage regions and deletions. We introduce CoVpipe2 to address the above challenges and have compared and successfully validated the pipeline against selected publicly available benchmark datasets. CoVpipe2 features high usability, reproducibility, and a modular design that specifically addresses the characteristics of short-read amplicon protocols but can also be used for whole-genome short-read sequencing data.

**Conclusions:** CoVpipe2 has seen multiple improvement cycles and is continuously maintained alongside frequently updated primer schemes and new developments in the scientific community. Our pipeline is easy to set up and use and can serve as a blueprint for other pathogens in the future due to its flexibility and modularity, providing a long-term perspective for continuous support. CoVpipe2 is written in Nextflow and is freely accessible from \href{https://github.com/rki-mf1/CoVpipe2}{github.com/rki-mf1/CoVpipe2} under the GPL3 license.

## Introduction

Since the publication of the first genome sequence of the novel SARS-CoV-2 virus in January 2020 – just 12 days after the initial report of the virus – the international GISAID database ^1^^–^^3^ now includes more than 15.5 million SARS-CoV-2 whole-genome sequences (accessed May 23, 2023). The genomic data and metadata collected in GISAID and other resources such as EBI’s COVID-19 Data Portal ^4^ are pivotal for the largest worldwide genomic surveillance effort ever undertaken to track the evolution and spread of the virus causing the COVID-19 disease. Important viral genome regions have been monitored for mutations, for example, in the spike gene and other immunologically relevant loci. The reconstruction of accurate SARS-CoV-2 genomic sequences is paramount to detect and track substitutions, insertions, and deletions correctly; interpret them in terms of vaccine development, test the efficiency of target regions and antibody binding sites, detect outbreaks and transmission chains, and finally inform public health authorities to consider adjustment of containment measures. ^5^

According to Ewan Birney, director of EMBL-EBI in Cambridge, U.K., “Genome sequencing is routine in the same way the U.S. Navy routinely lands planes on aircraft carriers. Yes, a good, organized crew does this routinely, but it is complex and surprisingly easy to screw up.”. ^6^

This quote is no less accurate for genome reconstruction, a crucial step in SARS-CoV-2 genomic surveillance. While sequencing efforts were scaling up rapidly around the globe, several pipelines for the reference-based assembly of SARS-CoV-2 genomes were developed in parallel and in an attempt to rapidly generate the necessary genome sequences ( Table 1). During the first lockdown in Germany in mid-March 2020, the Bioinformatics unit at the Robert Koch Institute (RKI), Germany’s Public Health institute, also started developing a genome reconstruction pipeline, specifically targeting Illumina amplicon sequencing data and amplicon schemes. During the development, the pipeline was extensively tested and has gone through continuous improvement due to adjusted wet lab protocols and primer schemes, to accurately call variants in low-coverage regions and near primer sites, to deal with deletions and low-coverage regions correctly, and to robustly reconstruct high-quality SARS-CoV-2 consensus sequences for downstream analyses and genomic surveillance. Due to these features and tests, CoVpipe1 was successfully used in the past at RKI’s sequencing facility and several labs nationwide. ^7^^–^^10^ If not addressed appropriately, genotyping errors can lead to wrong consensus sequences and thus impact downstream analyses such as phylogenetic reconstructions and transmission chain tracking in outbreaks. ^11^

**Table 1.** A collection of available software for SARS-CoV-2 genome reconstruction. Here, we mainly focus on open-source pipelines with available source code, specifically targeting the reconstruction of SARS-CoV-2 genomes, also including general pipelines adapted to work with SARS-CoV-2 sequencing data (e.g., V-pipe). Publ. – Publication focusing on the pipeline in a preprint or a peer-reviewed journal, Seq. tech. – focused sequencing technology, Implementation – main software backbone to run the tool (please note that not all pipelines use a workflow management system such as Nextflow ^25^ or Snakemake ^26^), Dependencies – a list of options to handle necessary software dependencies, Latest release – latest available release version as accessed May 23, 2023. mNGS – sequencing approach not based on amplicons but rather sequencing all available RNA/cDNA (metatranscriptomics/genomics).

Tool	Publ.	Code	Seq. tech.	Implementation	Dependencies	Latest release
**CoVpipe2**	–	github.com/rki-mf1/covpipe2	Illumina	Nextflow	Conda, Mamba, Docker, Singularity	0.4.2 (2023-06-09)
poreCov	^24^	github.com/replikation/poreCov	Nanopore	Nextflow	Docker, Singularity	1.8.3 (2023-05-12)
viralrecon	^27^	github.com/nf-core/viralrecon	Illumina, Nanopore	Nextflow	Conda, Docker, Singularity, Podman, Shifter, Charliecloud	2.6.0 (2023-03-23)
SIGNAL	^28^	github.com/jaleezyy/covid-19-signal	Illumina	Snakemake	Conda, Mamba, Docker (a single container with all dependencies)	1.6.2 (2023-05-20)
V-pipe	^29^	github.com/cbg-ethz/V-pipe	Illumina	Snakemake	Conda, Docker (a single container with all dependencies)	2.99.3 (2022-11-02)
NCBI SC2VC	–	github.com/ncbi/sars2variantcalling	Illumina, Nanopore, PacBio	Snakemake (multiple pipelines, run via wrapper script)	Docker (a single container with all dependencies)	3.3.4 (2022-10-28)
VirPipe	^30^	github.com/KijinKims/VirPipe	Illumina, Nanopore (mNGS)	Python, Nextflow	Conda and Docker	1.0.0 (2022-09-24)
ViralFlow	^31^	github.com/dezordi/ViralFlow	Illumina	Python	Conda, Singularity, Docker (one single container/environment)	v.0.0.6 (2021-04-18)
EDGE COVID-19	^32^	github.com/LANL-Bioinformatics/EDGE/tree/SARS-CoV2	Illumina, Nanopore	Python, Perl, available as web tool	Docker (a single container with all dependencies)	2.4.0 (2020-12-03)
Galaxy-COVID19	^33^	galaxyproject.org/projects/covid19/workflows	Illumina (amplicon & mNGS), Nanopore	Galaxy	Access to a Galaxy instance	different pipelines & versions
HAVoC	^34^	bitbucket.org/auto_cov_pipeline/havoc	Illumina	Shell scripts	Conda, Mamba	no release
PipeCoV	^35^	github.com/alvesrco/pipecov	Illumina (amplicon & mNGS)	Shell scripts	Docker	no release

While sequencing intensity and turnaround times on variant detection increased in different countries, there are also major disparities between high-, low- and middle-income countries in the SARS-CoV-2 global genomic surveillance efforts. ^12^ A recent study also found significant differences between bioinformatics approaches that use the same input data but detect different variants in SARS-CoV-2 samples. ^13^ In addition, each technology and sequencing approach has its own advantages and limitations, challenging a harmonized genomic surveillance of the virus. ^14^

In Germany, sequencing efforts increased tremendously in January 2021 following the entry into force of a federal directive (Coronavirus Surveillance Verordnung—CorSurV). Subsequently, a large-scale, decentralized genomic sequencing and data collection system (“Deutscher Elektronischer Sequenzdaten-Hub”, DESH) ^15^ has been established, accompanied by a medium-scale integrated molecular surveillance infrastructure (IMS-SC2) at the RKI. ^8^ By May 22, 2023, 1,227,036 whole-genome SARS-CoV-2 sequences that met the quality criteria were transmitted to the RKI via DESH. Due to their low cost, sensitivity, flexibility, specificity, and efficiency, amplicon-based sequencing designs are broadly used for SARS-CoV-2 sequencing and reference-based genome reconstruction. ^16^^–^^20^ From the ∼ 1,2 million DESH genomes (publicly available at github.com/robert-koch-institut/SARS-CoV-2-Sequenzdaten_aus_Deutschland), ∼ 1,1 million (90.91%) were sequenced with Illumina devices, highlighting the importance of the technology for genomic surveillance ( Table 2). Illumina technology has a lower share at the international level (78.57%), while Oxford Nanopore Technologies (ONT) increased to 12.73% ( Table 2). However, Illumina remains the most widely used approach among the various sequencing technologies, followed by ONT sequencing by a wide margin. A recent benchmark study also showed the advantages of using Illumina MiSeq compared to ONT GridION for SARS-CoV-2 sequencing, resulting in a higher number of consensus genomes classified by Nextclade ^21^ as good and mediocre. ^22^ However, these results are, of course, also dependent on the bioinformatics tool chains and could change as ONT becomes more accurate. ^23^ In addition, although both technologies require the same computational steps for reference-based genome reconstruction (preprocessing, mapping, variant calling, consensus), they need different tools optimized for either short- or long-read data and the associated error profiles to produce high-quality consensus sequences. Therefore, we developed one pipeline, especially for ONT data, ^24^ and CoVpipe2, specifically targeting high-accuracy SARS-CoV-2 amplicon data derived from short-read Illumina sequencing. CoVpipe2 is a Nextflow re-implementation of CoVpipe1 (written in Snakemake, gitlab.com/RKIBioinformaticsPipelines/ncov_minipipe) and comes with additional features, simplified installation, full container support, and continuous maintenance.

**Table 2.** Sequencing technologies used for SARS-CoV-2 sequencing of German and international samples. Data based on 1,2 million and 15,5 million whole-genome sequences submitted to the German DESH portal (“Deutscher Elektronischer Sequenzdaten-Hub”) and to the GISAID database, respectively (accessed: May 22, 2023). To the best of our knowledge, we corrected typos such as *Nanonopore*, *Nasnopore*, and *Illunima* in the GISAID metadata and summarized the available terms into the broader categories shown in this table ( *e.g.*, *NextSeq500* ⇒ *Illumina*). Entries we could not assign to one of the listed sequencing technologies were added to the *Other/Unknown* category. Please note that most German DESH sequences are also part of the GISAID data set. # – Number of sequences, % – Percentage of sequences on total data set.

Sequencing technology	# DESH	% DESH	# GISAID	% GISAID
**Illumina**	1,115,501	90.91	12,240,675	78.57
Nanopore	75,358	6.14	1,984,505	12.73
SMRT PacBio	0	0	573,214	3.67
Ion Torrent	29,450	2.40	349,001	2.24
MGI DNBSEQ	0	0	177,480	1.13
Sanger	0	0	57,820	0.37
Other/Unknown	6,727	0.54	195,833	1.25
Total	1,227,036	100	15,578,528	100

Here we present CoVpipe2, our pipeline engineered over nearly three years of pandemic genome sequencing that accurately reconstructs SARS-CoV-2 consensus sequences from Illumina short-read sequencing data, focusing on challenges associated with amplicon sequencing on a large scale. Besides implementation details, we also highlight pitfalls we discovered and solved during the pipeline development.

## Methods

### Implementation

CoVpipe2 is implemented using the workflow management system Nextflow ^25^ to achieve high reproducibility and performance on various platforms. The user can choose to use CoVpipe2 with Conda or Mamba support, ^36^ or containers (Docker, ^37^ Singularity ^38^) to handle all software dependencies. The Conda/Mamba environments and the container images are preconfigured and have fixed versions of the incorporated tools. The precompiled Docker containers are stored on hub.docker.com/u/rkimf1. Containers and environments are downloaded and cached automatically when executing CoVpipe2. If needed, the Docker images can also be converted into Singularity images by the pipeline. CovPipe2 includes Nextclade ^39^ and pangolin ^40^ for lineage assignment. Both tools rely on their latest code and database versions. To address this, we implemented the --update option inspired by poreCov, ^24^ which triggers an update to the latest available version from anaconda.org or hub.docker.com/u/rkimf1, respectively. --update is disabled by default; the tool versions can also be pinned manually.

When CoVpipe2 is running on a high-performance computing (HPC) cluster ( *e.g.*, SLURM, LSF) or in the cloud ( *e.g.*, AWS, GCP, Azure), all resources (CPUs, RAM) are pre-configured for all processes but can be customized via a user-specific configuration file. We use complete version control for CoVpipe2, from the workflow itself (releases) to each tool to guarantee reproducible results. To this end, all Conda/Mamba environments and containers use fixed versions. In addition, each CoVpipe2 release can be invoked and executed individually, and the tool versions used during genome reconstruction and analysis are listed in Nextflow report files.

CoVpipe2 is publicly available under a GPL-3.0 license at github.com/rki-mf1/covpipe2, where details about the implementation and different executions of CoVpipe2 can be found. We use GitHub’s CI for various pipeline tests, particularly a dry-run to check for integrity and an end-to-end test with special attention to the called variants to ensure continuous code quality and robust results.

### Operation

As a minimal setup, Nextflow (minimal version 22.10.1, nextflow.io) and either Conda, Mamba, Docker, or Singularity need to be installed for CoVpipe2. Nextflow can be used on any POSIX-compatible system, *e.g.*, Linux, OS X, and on Windows via the Windows Subsystem for Linux (WSL). Nextflow requires Bash 3.2 (or later) and Java 11 (or later, up to 18) to be installed. Initial installation and further updates to the workflow and included tools can be performed with simple commands:

# install (or update) the pipeline

nextflow pull rki-mf1/CoVpipe2

# check available pipeline versions

nextflow info rki-mf1/CoVpipe2

# run a certain release version

nextflow run rki-mf1/CoVpipe2 -r v0.4.1 --help

# test the installation with local execution and Conda

nextflow run rki-mf1/CoVpipe2 -r v0.4.1 -profile local,conda,test --cores 2 --max_cores 4

The pipeline can be executed on various platforms controlled via the Nextflow - profile parameter, which makes it easily scalable, *e.g.*, for execution on an HPC. Each run profile is created via combining different *Executors* (local, slurm) and *Engines* (conda, mamba, docker, singularity); the default execution-engine combination (profile) is -profile local, conda.

**Figure 1.**
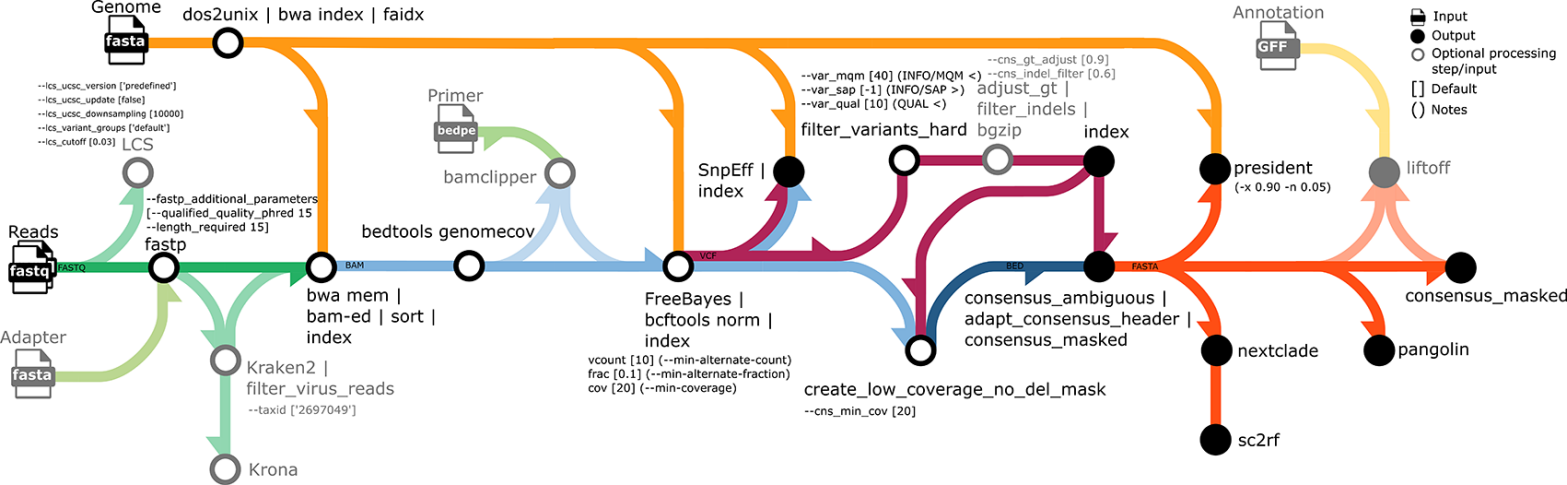
Overview of the CoVpipe2 workflow. The illustration shows all input ( 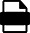) and output ( 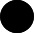) files as well as optional processing steps and optional input ( 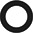). For each computational step, the used parameters and default values (in brackets [...]) are provided, as well as additional comments ((...)). The arrows connect all steps and are colored to distinguish different data processing steps: green – read (FASTQ) quality control and taxonomy filtering, yellow – reference genome (FASTA) and reference annotation (GFF) for lift-over, blue – mapping files (BAM) and low coverage filter (BED), purple – variant calls (VCF), orange – consensus sequence (FASTA). The icons and diagram components that make up the schematic figure were originally designed by James A. Fellow Yates and nf-core under a CCO license (public domain).

An overview of the workflow is given in Figure 1. FASTQ files and a reference genome sequence (FASTA) are the minimum required pipeline inputs. If no reference genome sequence is provided, the SARS-CoV-2 index case reference genome with accession number MN908947.3 (identical to NC_045512.2) is used by default (as well as the corresponding annotation GFF), and then only FASTQ files are required. All files can be provided via file paths or defined via a comma-separated sample sheet (CSV); thus, CoVpipe2 can run in batch mode and analyze multiple samples in one run. Optionally, raw reads are checked for mixed samples with the tool LCS. ^41^ Next, raw reads are quality-filtered and trimmed using fastp ^42^ and optionally filtered taxonomically by Kraken2. ^43^ We provide an automated download of a precalculated Kraken2 database composed of SARS-CoV-2 and human genomes from Zenodo. However, the user is free to use a custom database. The reads are aligned to the reference genome using BWA, ^44^ and the genome coverage is calculated by BEDTools genomecov. ^45^ Primers can be optionally clipped after mapping with BAMclipper, ^46^ which is essential to avoid contaminating primer sequences in amplicon data. To locate the primer sequences, a browser extensible data paired-end (BEDPE) file containing all primer coordinates is required as input. If only a BED file is provided, CoVpipe2 can automatically convert it to a BEDPE file. The user can also choose from provided popular VarSkip ( github.com/nebiolabs/VarSkip) and ARTIC primer schemes. ^47^ Next, FreeBayes ^48^ calls variants (default thresholds: minimum alternate count of 10, minimum alternate fraction of 0.1, and minimum coverage of 20), which are normalized with BCFtools norm. ^49^ Resulting variants are analyzed and annotated with SnpEff, ^50^ filtered by QUAL (default 10), INFO/SAP (default disabled), and INFO/MQM (default 40) values, and optionally adjusted for the genotype (default enabled with minimum variant frequency 0.9). Thus, in the case of mixed variants, the predominant variant can be defined as a homozygous genotype if its frequency reaches a certain threshold. With these settings, alternate variants with a frequency of more than 90% are set as alternative nucleotides. Furthermore, CoVpipe2 filters out indels below a certain allele frequency, which is, by default, enabled with a minimum allele frequency of 0.6. We create a low coverage mask, without deletions, from the mapping file and the adjusted and filtered VCF file (default coverage cutoff 20), which is then used in the consensus calling with BCFtools consensus. We output an ambiguous consensus sequence with IUPAC characters ^51^ (RYMKSWHBVDN) and a masked one only containing ACTG and Ns. Then, PRESIDENT ( GitHub) assesses the quality of each reconstructed genome via pairwise alignment to the SARS-CoV-2 index case (NC_045512.2) using pblat ^52^ with an identity threshold of 0.9 and N threshold of 0.05 per default. pangolin assigns a lineage and Nextclade annotates the mutations; the Nextclade alignment serves as input for sc2rf (original repository from github.com/lenaschimmel/sc2rf, with updates from github.com/ktmeaton/ncov-recombinant) for detection of recombinants. If an annotation file is provided, the reference annotation is mapped to the reconstructed genome with Liftoff. ^53^

All results are summarized via an R Markdown template. The resulting HTML-based report summarizes different quality measures and mapping statistics for each input sample, thus allowing the user to spot low-performing samples even in extensive sequencing runs with many samples. A conditional notification warns the user if samples identified as negative controls (by matching the string ’NK’) show high reference genome coverage (threshold over 0.2).

**Common challenges in amplicon-based genome reconstruction and solutions in CoVpipe2**

Here, we highlight some implementation decisions that prevent common pitfalls and thus improve the quality of reconstructed SARS-CoV-2 genomes. Although amplicon-based approaches are widely used, these technologies are associated with flaws and limitations that must be considered to ensure that the genotypes obtained, and thus the resulting genome sequences, are reliable. ^13^^,^^54^ While some of these implementations might seem obvious and easy to fix, we frequently observed specific errors in the vast amount of consensus genome sequences sent to us via the DESH system.

**Figure 2.**
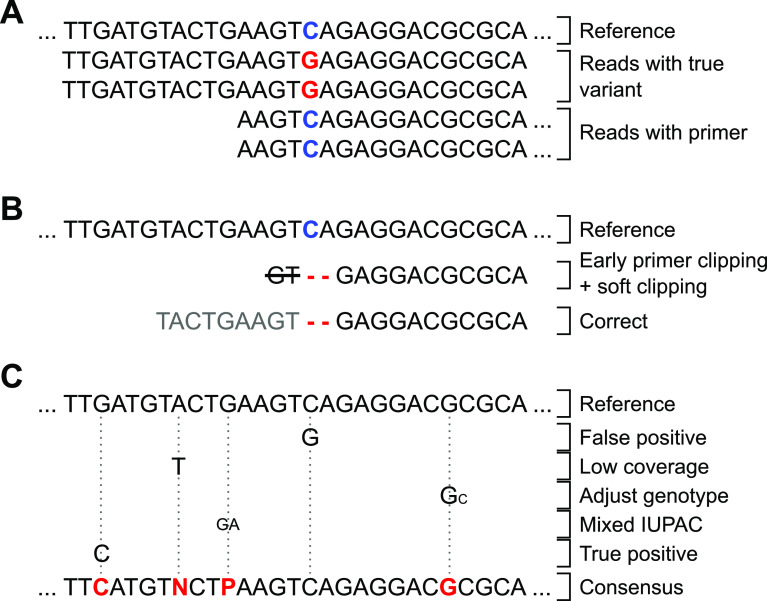
Exemplary cases that should be considered during genotyping. (A) Primer sequences need clipping to call true variants (red) and not mask them via reference bases (blue). (B) Early primer clipping may result in missed deletions due to algorithmic soft clipping (primer sequence in light gray). (C) Genotyping parameters must be carefully set to reliably call different variant cases and represent them in the final consensus. CoVpipe2 puts primer clipping after the mapping step to prevent B) and implements carefully chosen default parameters for robust genotyping also of mixed variants, C).

**Accurate primer clipping to avoid dilution and edge effects** Primer clipping is an essential step for amplicon sequencing data because primers are inherent to the reference sequence and can disguise true variants in the sample. ^13^ However, removing primers before the mapping step can result in unwanted edge effects. ^55^ For example, deletions located close to the end of amplicons may be soft-clipped by the mapping software and hence can not be called as variants subsequently ( Figure 2). Therefore, primer clipping should be performed after mapping to prevent any soft clipping of variants close to the amplicon ends.

As an example and worst-case scenario, clipping primer sequences before mapping bears the risk of missing a critical deletion used to define the previous VOC B.1.1.7, namely deletion HV69/70 in the spike gene (S:H69-, S:V70-). We observed such a misclassification using Paragon CleanPlex amplicon-based sequencing. The kit uses primers similar to the ARTIC protocol V3, where the deletion S:HV69/70 is close to the end of an amplicon. If primer clipping is performed before mapping, the mapping tool might soft clip the amplicon end rather than opening a gap, which is more expensive than masking a few nucleotides ( Figure 2). We checked 151,565 B.1.1.7 sequences obtained via DESH for the characteristic S:H69, S:V70 deletions and found that 139,891 (92.3%) included the deletion. The remaining sequences contained the deletion only partially or lacked it completely. It is unlikely that these B.1.1.7 sequences lost this characteristic deletion. Thus we assume that some of the reconstructed Alpha sequences sent to the RKI from different laboratories in Germany do not account for the described effect and thus miss the detection of this deletion. Unfortunately, we don’t know which bioinformatics pipelines the submitting laboratories were using and can only assume an error because of missing or incorrect primer clipping. To avoid this problem, we shift primer clipping after the read mapping step in CoVpipe2; otherwise, a vital feature of an emerging virus lineage might be missed if mutations accumulate close to amplicon ends.

**Genotype adjustment to exclude sporadic variant calls** As an additional feature, we provide the option to adjust the genotype for sites where the vast majority of reads support a variant call, but the variant was called heterozygous. By default, the genotype of these locations is set to homozygous if a particular variant call is supported by 90% of the aligned reads.

**Deletion-aware masking of low-coverage regions** We ensure that only low-coverage positions that are not deletions are masked. Several pipelines implement a feature to mask low-coverage regions. However, deletions are basically genomic regions with no coverage, and if not appropriately implemented, a pipeline might accidentally mask deletions as low-coverage regions. To prevent this, CoVpipe2 creates a low coverage mask (default minimum coverage 20) from the BAM file with BEDtools genomecov. In the second step, BEDtools subtract removes all deleted sites from the low-coverage mask. Finally, the mask is used in the consensus generation with BCFtools consensus.

**IUPAC consensus generation with indel filter** CoVpipe2 generates different consensus sequences based on the IUPAC code. First, an explicit consensus is generated where all ambiguous sites and low-covered regions are hard-masked. Second, a consensus where only low-covered regions are hard-masked. The pipeline includes as much information as possible in the unambiguous consensus sequence by adding low-frequency variants with the respective IUPAC symbol. However, no symbol represents “indel or nucleotide”, so indels are always incorporated into the consensus sequence. As a result, low-frequency or heterozygote indels, often false positives, can introduce frameshifts into the consensus sequence. We overcome this with an indel filter based on allele frequency before consensus generation. Thus, indels below a defined threshold (per default 0.6) are not incorporated in the consensus sequences but can still be looked up in the VCF file.

**Additional features beyond genome reconstruction**

Over time, we added features beyond genome reconstruction to CoVpipe2 to answer newly emerging questions during the pandemic. The modular design of our implementation makes this seamless.

**Mixed infections and recombinants** We included LCS for raw reads. LCS was originally developed for the SARS-CoV-2 lineage decomposition of mixed samples, such as wastewater or environmental samples. ^41^ In the context of amplicon-based SARS-CoV-2 sequencing, we use LCS results for indications for potential new recombinants, and possible mixed infections (co-infections with different SARS-CoV-2 lineages).

Further, we added sc2rf to detect potential new recombinants via screening the consensus genome sequences. Like other tools and scripts, sc2rf depends on up-to-date lineage and mutation information, specifically, on a manually curated JSON file. We switched to another repository ( JSON GitHub project) than the original one that includes the sc2rf scripts because of more frequent updates on the JSON file.

**Keeping up to date with lineage and clade assignments** We implemented an update feature for pangolin and Nextclade. Both tools, especially pangolin, rely on the latest datasets for the lineage assignment of newly designated Pango lineages. ^56^ Depending on the selected engine, Conda/Mamba or container execution, CoVpipe2 checks for the latest available version from Anaconda or our DockerHub, respectively. The tool versions can also be pinned manually.

**Prediction of mutation effects** The called variants are annotated and classified based on predicted effects on annotated genes with SnpEff. SnpEff reports different effects, including synonymous or non-synonymous SNPs, start codon gains or losses, stop codon gains or losses; and classifies them based on their genomic locations. Lastly, CoVpipe2 uses Liftoff to generate an annotation for each sample if a reference annotation is provided.

## Results

### Selection of benchmark datasets

We compared the results of CoVpipe2 (v0.4.0) with publicly available benchmark datasets for SARS-CoV-2 surveillance ^57^ ( GitHub CDC data). For our study, we selected from the available benchmark datasets all samples that were sequenced with the ARTIC V3 primer set (according to CDC data, release v0.7.2), ending up with in total 54 samples from three datasets:

• i) 16 samples from VOI/VOC lineages (dataset 4)
• ii) 33 samples from non-VOI/VOC lineages (dataset 5)
• iii) 5 samples from failed QC (dataset 6)

We stick to the naming scheme (dataset 4, 5, and 6) as declared in the original study. ^57^ For all 54 samples, we downloaded the raw reads from ENA with nf-core/fetchngs ( v1.9). ^58^ For 49 samples, we downloaded the available consensus sequences from GISAID ^1^^–^^3^ (samples from dataset 4 and 5). We run CoVpipe2 with Singularity, species filtering ( --kraken) and the latest pangolin and Nextclade versions at that point (containers: rkimf1/pangolin:4.2-1.19--dec5681, rkimf1/nextclade2:2.13.1--ddb9e60). We further examined the consensus sequences from dataset 4 and 5, comparing CoVpipe2 and GISAID sequences: We compared lineages information of the reconstructed genomes assigned by pangolin with the indicated lineages from CDC data. Note that the pangolin versions and datasets differ. Also, we run Nextclade (same container version as noted above) to compare the mutation profile, and MAFFT, ^59^ v7.515 (2023/Jan/15), for comparison on sequence level of the unambiguous IUPAC consensus of CoVpipe2.

### Reporting

**Figure 3.**
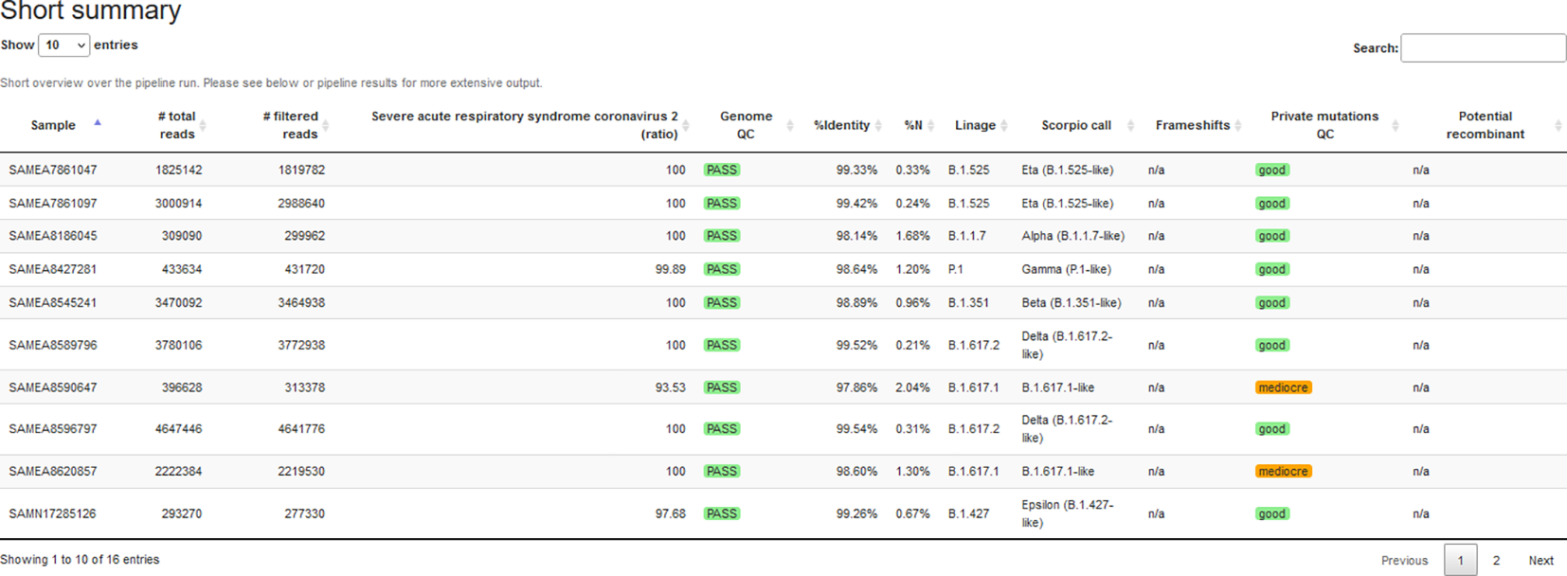
Extract of CoVpipe2’s summary report for dataset 4. The standalone HTML report summarizes different quality measures and tool results. The overview table can include a conditional notification for negative controls with high reference genome coverage (not shown in this example). All tables are searchable and sortable.

The HTML report consists of several sections and summarizes different results. The first table aggregates critical features for each sample: the number of reads, genome QC, the assigned lineage, and recombination potential, see Figure 3. Furthermore, read properties such as the number of bases and the length of reads (before/after trimming) are summarized. An optional table lists the species filtering results emitted by Kraken2. ^43^ The report summarizes reads mapped to the reference genome (number and fraction of input) and the median/standard deviation of fragment sizes, shown as a histogram plot. Genome-wide coverage plots allow users to observe low-coverage regions and potential amplicon drop-outs to optimize primers. We summarize the output for all samples in different tables: i) genome quality from PRESIDENT output, ii) lineage assignments from pangolin output, and iii) variants in amino acid coordinates and detected frameshifts from Nextclade output.

## Discussion

### CovPipe2 reconstructs consensus genomes matching previously reported SARS-CoV-2 lineages

Here, we compare the results of CoVpipe2 against a selection of available benchmark datasets ^57^ and their respective consensus genome sequences available from GISAID. ^1^^–^^3^ We discuss the observed differences. No software is perfect, and CoVpipe2 may have problems with certain combinations of amplicon schemes and sequencing designs, leading to specific borderline cases in variant detection, which we also highlight and discuss. In addition, in conflicting cases, the real sequence often stays unknown until further sequencing efforts are performed. This makes continuous development and testing of bioinformatics pipelines all the more critical.

**Dataset 4, VOI/VOC lineages**

**Lineages** Despite the different pangolin tool versions and lineage definitions that changed over time, all pangolin lineages from CoVpipe2 match the corresponding lineages reported at github.com/CDCgov/datasets-sars-cov-2, see *Extended data*, Table S1.

**Pairwise alignment** The sequence identity ranges from 98.44 % to 99.79 % (including Ns) between the corresponding GISAID and CoVpipe2 genome pairs ( *Extended data*, Table S2). Nine out of 16 sequences are identical when mismatches resulting from gaps are not considered. For one sample, the corresponding GISAID and CoVpipe2 sequences contain three ACGT-nucleotide mismatches. Seven out of 16 GISAID consensus sequences do not contain any Ns, possibly indicating that low coverage regions have been not masked. The respective reconstructed genomes from CoVpipe2 contain Ns located in the first or last 150 nucleotides of the genome sequences, thus masking low coverage regions ( *Extended data*, Table S3). Due to tiled PCR amplicons, 5′ and 3′ ends of the genome usually have too little coverage or are not sequenced. The genome ends containing Ns seem to be trimmed in 14 GISAID genomes. Overall, the number of Ns in the GISAID and CoVpipe2 genomes is comparable, with CoVpipe2 genomes tending to have more Ns due to the low coverage filter resulting in Ns at genome ends. In addition, CoVpipe2 does not trim Ns from the genome ends and might be more conservative with its default settings, as positions below 20 X coverage ( --cns_min_cov) will be masked with ambiguous N bases in the consensus sequence.

**Mutations** Although all genomes reconstructed from CoVpipe2 were assigned to the same lineage as previously reported ( CDC data), there are minor differences in Nextclade’s mutation profile, *Extended data*, Table S4. Three of 16 reconstructed genomes have one or two nucleotide substitutions more than the respective GISAID genome.

**Dataset 5, non-VOI/VOC lineages**

**Lineages** Pangolin lineages from CoVpipe2 exactly match the reported lineages at CDC data in 27 of 33 samples, see *Extended data*, Table S5. For five samples, the pangolin lineage based on the genome sequence reconstructed by Covpipe2 is the parent lineage of the expected sub-lineage. For example, the genome sequence of sample SAMN15919634 was assigned to the B.1.1 lineage after CoVpipe2 reconstruction, whereas the corresponding GISAID sequence is assigned to B.1.1.431 ( CDC data). Since the lineage assignments of Nextclade exactly match for each CoVpip2-GISAD pair, the different pangolin and pangolin data versions used in CoVpipe2 and Xiaoli and Hagey *et al*. ^57^ are most likely the reason for this discrepancy. Different parameter thresholds can also lead to different lineage assignments. For example, the genome sequence of SAMN17571193 was assigned to B.1.1.450 by CoVpipe2 compared to B.1.1.391 in Xiaoli and Hagey *et al*. ^57^ The mutation profile differs by two additional SNPs (genome coordinates: C3037T and C3787T) constituting one mutation on amino acid level (ORF1a:D1962E) in the GISAID consensus sequence. Both positions (3037 and 3787) have a read coverage below 20, so they are not eligible for variant calling with default settings in CoVpipe2 and are masked with N. Therefore, this difference in lineage assignment is due to CoVpipe2’s more restrictive approach to integrating the called variants into the consensus.

**Pairwise alignment** The pairwise sequence identity ranges from 95.96 % to 99.80 % (including Ns) between the GISAID and CoVpipe2’s reconstructed genome sequences ( *Extended data*, Table S6). Ignoring gap mismatches, 13 out of 33 sequences are identical. No sample contains ACGT mismatches. 18 out of 33 GISAID consensus sequences do not contain any Ns. Sixteen of the respective reconstructed genomes have Ns, all located in the first or last 150 nucleotides ( *Extended data*, Table S7). Because of tiled PCR amplicons, the 5′ and 3′ ends typically have too little coverage or are not sequenced. The 5′ and 3′ genome ends containing Ns seem trimmed in 30 GISAID genomes. Overall, the number of Ns in the GISAID and CoVpipe2 genomes is comparable, with CoVpipe2 genomes tending to have more Ns. CoVpipe2 does not trim Ns from the genome ends and might be more conservative with its default settings, as positions below 20 ( --cns_min_cov) will be masked with N in the consensus sequence.

**Mutations** There are minor differences in Nextclade’s mutation profile ( *Extended data*, Table S8). Five of the 33 reconstructed genomes have one or two more nucleotide substitutions compared to the GISAID genome.

**Dataset 6, samples failing quality control**

For the five samples from the failed QC dataset by Xiaoli and Hagey *et al*., ^57^ CoVpipe2 correctly labeled four samples with failed genome QC. Three samples contain at least one frameshift, whereas two samples, SAMN17486862 and SAMN17822806, were reconstructed by CoVpipe2 without frameshifts ( *Extended data*, Table S9). The consensus sequence of sample SAMN17486862 passed QC according to CoVpipe2’s genome QC criteria.

All the selected samples from this dataset have originally failed QC because of a VADR ^60^ alert number greater than one. ^57^ VADR is part of the TheiaCoV (formerly ‘Titan’) 1.4.4 pipeline, ^61^ which was used to analyze the samples in Xiaoli and Hagey *et al*. ^57^ Among other things, VADR considers frameshifts, which do not occur in two genomes reconstructed with CoVpipe2. However, one of these two consensus genomes (SAMN17822806) contains too many Ns to pass CoVpipe2’s genome QC.

### Limitations

The nature of a computational pipeline is that it is a chain of existing individual tools. Especially given the rapid evolution of SARS-CoV-2 during the pandemic, many reference-based tools rely on up-to-date databases and resources to reflect the current situation. For example, LCS depends on a variant marker table and user-defined variant groups. Similarly, sc2rf relies on a list of common variants for each lineage. In addition, Nextclade and pangolin periodically publish up-to-date datasets. Therefore, it is critical for a pipeline, especially in the context of a surveillance tool for rapidly evolving pathogens such as SARS-CoV-2, to allow for regular updates to the underlying data structures. While we have implemented some functionality to update tools such as Nextclade and pangolin automatically, this is not possible for all resources and can only be achieved through the continuous development and maintenance of a pipeline. Furthermore, our default settings may not fit all input data and must be selected carefully.

Finally, there must be enough good-quality input reads to reconstruct a genome successfully. In particular, with amplicon sequencing data, some regions might have a lower coverage due to amplicon dropouts. Thus, the genome as a whole can be reconstructed with acceptable quality. However, some essential mutations can be missing due to low coverage or variant-calling quality.

## Conclusions

Accurate and high-throughput genotyping and genome reconstruction methods are central for monitoring SARS-CoV-2 transmission and evolution. CoVpipe2 provides a fully automated, flexible, modular, and reproducible workflow for reference-based variant calling and genome reconstruction from short-read sequencing data, emphasizing amplicon-based sequencing schemes. Due to the implementation in the Nextflow framework, the setup and automatic installation of the required tools and dependencies is simple and allows the execution on different computing platforms. The comparison with a benchmark dataset showed comparable results where differences could be pinned down to different parameters, filtering thresholds, and tool versions used for lineage assignments. The pipeline is optimized for SARS-CoV-2 amplicon data but can also be used for other viruses and whole-genome sequencing protocols. Amplicon-optimized default parameters and the ability to customize critical parameters, combined with comprehensive reporting, ensure the quality of reported genomes and prevent the inclusion of low-quality sequences in downstream analyses and public repositories. Furthermore, CoVpipe2 will form the basis for additional genomic surveillance programs at the Public Health Institute of Germany that will extend to other viruses.

## Data Availability

All SRA accession IDs of raw reads and GISAID IDs of consensus sequences are listed at ^57^ and github.com/CDCgov/datasets-sars-cov-2; and in our Open Science Framework repository osf.io/26hyx ( *Extended data*: https://doi.org/10.17605/OSF.IO/MJ6EQ). ^62^ We used ARTIC V3 samples from dataset 4 (VOI/VOC lineages, 16 samples), dataset 5 (non-VOI/VOC lineages, 33 samples), and dataset 6 (failedQC, 5 samples) from the original study of Xiaoli and Hagey *et al*. ^57^ Open Science Framework. Lessons learned: overcoming common challenges in reconstructing the SARS-CoV-2 genome from short-read sequencing data via CoVpipe2, https://doi.org/10.17605/OSF.IO/MJ6EQ. ^62^ This project contains the following extended data:
• Data folder Accession ID. (SRA and GISAID accession ID lists) • Data folder Comparison. (Scripts and results of the benchmark comparison) • Data folder CoVpipe2 results. (Results of CoVpipe2 for each benchmark dataset) • Data folder Extended data. (Supplementary tables S1-S9)

## Software availability

• Software and source code available from: https://github.com/rki-mf1/covpipe2
• Archived source code at time of publication: https://doi.org/10.5281/zenodo.8082695. ^63^
• License: GNU General Public License v3.0 (GPL3)
