# Supplementary material for: Lessons learned: overcoming common challenges in reconstructing the SARS-CoV-2 genome from short-read sequencing data via CoVpipe2
Source: F1000Res. 2024 Apr 16;12:1091. Originally published 2023 Sep 1. [Version 2] doi: 10.12688/f1000research.136683.2 (PMC11074694; doi:10.12688/f1000research.136683.2)
Supplement: Peer review history

# Reviewer report: Reviewer response for version 2

**DOI:** 10.5256/f1000research.164888.r266843

**Published:** 2024-05-06

**Authors:** Sondes Haddad-Boubaker

**Affiliations:** 1 University of Tunis El Manar, Tunis, Tunisia

The revision made is appropriate thus I approve of the paper in the current form.

 Thank you for publishing and sharing results.

Are the conclusions about the tool and its performance adequately supported by the findings presented in the article?

Partly

Is the rationale for developing the new software tool clearly explained?

Yes

Is the description of the software tool technically sound?

Yes

Are sufficient details of the code, methods and analysis (if applicable) provided to allow replication of the software development and its use by others?

Partly

Is sufficient information provided to allow interpretation of the expected output datasets and any results generated using the tool?

Partly

Reviewer Expertise:

Virology

I confirm that I have read this submission and believe that I have an appropriate level of expertise to confirm that it is of an acceptable scientific standard.

# Reviewer report: Reviewer response for version 1

**DOI:** 10.5256/f1000research.149827.r233668

**Published:** 2024-01-17

**Authors:** Sondes Haddad-Boubaker

**Affiliations:** 1 University of Tunis El Manar, Tunis, Tunisia

This paper presents a comprehensive bioinformatics workflow designed for the reconstruction of SARS-CoV-2 genomes using short-read sequencing data. The workflow description offers a detailed overview of the processes involved; however, there are opportunities for improvement to enhance the overall quality of the paper.

 1-Organization:

 While the overall structure is clear, introducing numbering at both the heading and sub-heading levels would enhance clarity and facilitate better distinction between various steps in the workflow.

 2-Technical Terminology:

 Given the diverse audience, including virologists and other biologists, it is crucial to ensure accessibility by providing clear definitions for all technical terms and acronyms. This will make the paper more easy to readers who may not be familiar with specific bioinformatics or genomics terminology.

 3- References and Sources:

 Include proper references and sources for the tools and databases mentioned by providing specific URLs for easy access to external resources.

 4-Methods and Results:

 - Introduce a dedicated "Pipeline Evaluation" subsection in both the "Methods" and "Results" sections and change the abstract section accordingly.

 -Include details on the investigated sequences, with a thorough description of sample types, cycle threshold (ct) values, and sample categories.

 -Consider expanding the evaluation by incorporating additional samples/sequences, especially failed sequences from samples with varying ct values (especially high ct values). The Investigation of the contribution of the pipeline in obtaining reliable sequences from samples with high ct values may be helpfull  for virologist who are dealing with such challenges especially in samples obtained from long-term excretors.

 - Please Illustrate "common challenges" with a graph or figure for better presentation and understanding.

 5- Discussion:

 - Emphasize and discuss the added value of the pipeline in obtaining reliable sequences from samples with high ct values. Compare the results obtained using this pipeline with those from other existing pipelines to highlight its superiority.

 - Provide detailed insights into how this pipeline can be applied to study other viruses,

Are the conclusions about the tool and its performance adequately supported by the findings presented in the article?

Partly

Is the rationale for developing the new software tool clearly explained?

Yes

Is the description of the software tool technically sound?

Yes

Are sufficient details of the code, methods and analysis (if applicable) provided to allow replication of the software development and its use by others?

Partly

Is sufficient information provided to allow interpretation of the expected output datasets and any results generated using the tool?

Partly

Reviewer Expertise:

Virology

I confirm that I have read this submission and believe that I have an appropriate level of expertise to confirm that it is of an acceptable scientific standard, however I have significant reservations, as outlined above.

# Response

**Published:** 2024-04-10

**Authors:** Martin Hölzer

**Affiliations:** Genome Competence Center (MF1), Robert Koch Institute, Berlin, Berlin, Germany

(I) - ***Organization:***

 [1] Reviewer Concern: *While the overall structure is clear, introducing numbering at both the heading and sub-heading levels would enhance clarity and facilitate better distinction between various steps in the workflow.*

 Author Response: Thanks for the suggestion. Numbering (sub)sections helps to structure the text better and distinguish which parts belong together semantically. However, there is little we can do about it, as this is the journal's style. Nevertheless, we will ask the editor/typesetting team if that’s possible.  

 --------------------------------------------------------------------------------------------------------------------

 (II) - ***Technical Terminology:***

 [2] Reviewer Concern: *Given the diverse audience, including virologists and other biologists, it is crucial to ensure accessibility by providing clear definitions for all technical terms and acronyms. This will make the paper more easy to readers who may not be familiar with specific bioinformatics or genomics terminology.*

 Author Response: Thanks for the comment. We agreed and added a list of Abbreviations to the manuscript to make it easier for readers to follow the story. 

- COVID-19 - Coronavirus disease 2019
- SARS-CoV-2 - Severe acute respiratory syndrome coronavirus 2
- GPL3 license - GNU General Public License
- GISAID - Global Initiative on Sharing All Influenza Data
- EBI - European Bioinformatics Institute
- EMBL - European Molecular Biology Laboratory
- RKI - Robert Koch Institute
- CorSurV - Coronavirus Surveillance Verordnung (eng., Coronavirus Surveillance Regulation)
- DESH - Deutscher Elektronischer Sequenzdaten-Hub (eng., German Electronic Sequence Data Hub)
- IMS-SC2 - Integrated Molecular Surveillance for SARS-CoV-2
- ONT - Oxford Nanopore Technologies
- NGS - Next-Generation Sequencing
- HPC - High-Performance Computing
- WSL - Windows Subsystem for Linux
- CSV  file - Comma-Separated Values file
- GFF file - General Feature Format file
- BEDPE file - Browser Extensible Data Paired-End file
- VCF file - Variant Call Format file
- HTML - Hypertext Markup Language
- BAM file -  Binary Alignment and Map file
- BED file - Browser Extensible Data file
- CCO license - Creative Commons Zero license 
- VOC - Variants of Concern
- VOI - Variants of Interest
- IUPAC -  International Union of Pure and Applied Chemistry
- indel - Insertion/Deletion Variant
- JSON file - JavaScript Object Notation file
- CDC - Centers for Disease Control and Prevention
- ENA - European Nucleotide Archive
- QC - Quality Control
- PCR - Polymerase Chain Reaction
- ID - Identifier
- SRA - Sequence Read Archive

--------------------------------------------------------------------------------------------------------------------

 (III) - ***References and Sources***

 [3] **Reviewer Concern:** *Include proper references and sources for the tools and databases mentioned by providing specific URLs for easy access to external resources.*

**Author Response: **We agree that it’s crucial to acknowledge all tools and resources properly. When there is an original publication for a tool or database, we cite the publication. If not, we cite the code repository or the URL to the resource (such as GitHub or Zenodo). We carefully checked the text again and added citations/URLs if they were missing and necessary. For example, Rev #1 also commented that the specific URL to the custom Kraken 2 database on Zenodo was missing. We added that.

 --------------------------------------------------------------------------------------------------------------------

 (IV) - ***Methods and Results***

 [4] **Reviewer Concern**: *Introduce a dedicated "Pipeline Evaluation" subsection in both the "Methods" and "Results" sections and change the abstract section accordingly.*

**Author Response:** We fully agree that presenting the implementation of a pipeline together with its evaluation with additional analysis can be confusing. So what are the "Methods" and the "Results" parts, then? This is often a problem when simultaneously presenting and evaluating a new software implementation. But we also need to adhere to the style guidelines for journals.

 We wrote a “Software Tool Articles” and have to follow this structure: https://f1000research.com/for-authors/article-guidelines/software-tool-articles .

 As you can see in these guidelines, “Software Tool Articles typically contain the following sections: Introduction, Methods, Results (Optional), Use Cases (Optional), Conclusions/Discussion.” In the first version, we skipped the “Use Cases” section to discuss our example data sets for pipeline evaluation directly in the “Results” section. However, thanks to your comment, we believe that also skipping the “Results” section entirely makes our manuscript clearer.  We deleted the “Results” section and added the subsections “Selection of benchmark datasets and pipeline evaluation” and “Reporting” at the end of the “Methods”. We changed the subsection “Selection of benchmark datasets” to “Selection of benchmark datasets and pipeline evaluation” to include your suggestion. 

 We think that the structure is now clearer because we first describe in the “Methods” the implementation of the pipeline and how to operate it, according to the journal guidelines for “Software Tool Articles”: The Methods should “Include a subsection on **Implementation** describing how the tool works and any relevant technical details required for implementation; and a subsection on **Operation**, which should include the minimal system requirements needed to run the software and an overview of the workflow.” 

 Then, we describe some specific implementation decisions followed by the example data sets for pipeline evaluation and, finally, the report structure we implemented.

 According to the journal guideline for “Software Tool Articles”: “Abstracts are structured into Background, Methods, Results, and Conclusions”, thus, we can not change sections in the abstract. 

 Although we generally agree that different subheadings would help follow the story, we must also stick to the journal guidelines. 

 --------------------------------------------------------------------------------------------------------------------

 [5] **Reviewer Concern**: *Include details on the investigated sequences, with a thorough description of sample types, cycle threshold (ct) values, and sample categories.*

**Author Response: **We only use publicly available data sets and reference the original sources (publication, GitHub, and ENA repositories). We suggest that readers should refer to the original sources for further details. However, the description of the benchmark datasets is now also part of the “Methods”. Here, we describe:

 “We compared the results of CoVpipe2 (v0.4.0) with publicly available benchmark datasets for SARS-CoV-2 surveillance 57 ( GitHub CDC data ).”

 [57] Xiaoli L, Hagey JV, Park DJ, et al.: Benchmark datasets for sars-cov-2 surveillance bioinformatics. PeerJ. 2022;10:e13821. 10.7717/peerj.13821

 We do not want to mirror the details of the benchmark data sets that are described in the original sources. In addition, we can not provide additional information, such as Ct values, because, to the best of our knowledge, this information is not available in the original publication or in the data source (GitHub, ENA). 

 --------------------------------------------------------------------------------------------------------------------

 [6] **Reviewer Concern**: *Consider expanding the evaluation by incorporating additional samples/sequences, especially failed sequences from samples with varying ct values (especially high ct values). The Investigation of the contribution of the pipeline in obtaining reliable sequences from samples with high ct values may be helpful  for virologist who are dealing with such challenges especially in samples obtained from long-term excretors.*

**Author Response: **Thanks for the comment. We agree that investigating challenging samples is especially interesting for users of CoVpipe2. As described above (Q [5]), we selected a publicly available benchmark dataset for SARS-CoV-2 surveillance (Xiaoli et al. 2022) to compare our results directly with previous calculations. In addition, this data set also includes difficult samples that should not withstand automatic quality control (QC) and could mimic high Ct values. 

 CoVpipe2 was developed as a robust and standardized workflow to support genome reconstruction in genomic surveillance programs. Thus, our main goal in developing CoVpipe2 was to provide a robust bioinformatics pipeline for short-read sequencing data that recognizes important mutations with decent allele frequency and automatically identifies and masks ambiguous positions. We implemented parameters (20X coverage to consider a position for variant calling, 90% ACGT nucleotide identity to the reference) to discover low-quality samples that might originate from high Ct values. Running the pipeline on amplicon sequencing data from samples with high Ct can result in "read stacks" with high sequence depth for certain well-amplified amplicons, but it could also lead to low horizontal genome coverage due to low input RNA quantity. CoVpipe2 will report such samples as “failed” in the QC report. Thus, only samples with a decent vertical (sequencing depth) and horizontal genome coverage should be used for downstream genomic surveillance and trustworthy lineage assignment. 

 Nevertheless, CoVpipe2 also reports the full intermediate results, such as BAM files and unfiltered VCF files. Experienced users can investigate all variant calls and their respective allele frequencies - also for QC-failed samples. Thus, it is also possible to investigate mixed variant calls (co-infection, recombinants) and low-frequency variants with the help of CoVpipe2. However, for routine genomic surveillance applications, such challenging samples will be automatically flagged as QC-failed in the pipeline, supporting non-expert users in decision-making and selecting suitable samples for surveillance. Obtaining reliable consensus genomes from high Ct samples is generally difficult. In our experience, it is better to flag such samples with a warning and inform the user that those are of lower quality and probably not suited for further downstream analysis. Thus, CoVpipe2 helps virologists identify such challenging samples so that they can be selected for re-sequencing or exclusion from downstream analysis.  

 --------------------------------------------------------------------------------------------------------------------

 [7] **Reviewer Concern**: *Please Illustrate "common challenges" with a graph or figure for better presentation and understanding.*

**Author Response: ** Here, we present a bioinformatics pipeline with the specific objective of reconstructing robust SARS-CoV-2 consensus genomes from patient samples and short-read data, which is also reflected in the title of our paper. We present CoVpipe2 as a solution to overcome such challenges in reconstructing robust SARS-CoV-2 genomes from short-read (amplicon) data. In Figure 2, we explicitly illustrate common challenges regarding variant calling, which is one of the main obstacles in many reference-based virus bioinformatics pipelines, and where we specifically integrated solutions in CoVpipe2 to overcome such challenges. Besides the dedicated figure for variant calling challenges, we examine other relevant challenges in the context of the CoVpipe2 implementation, such as amplicon drop-outs, in the text.

 For a general overview, we think that common challenges in the context of amplicon sequencing and virus bioinformatics need to be more broadly addressed in dedicated benchmark studies such as those already available from Beerenwinkel et al. 2012; Murray et al. 2015; Fitzpatrick et al. 2022; Liu et al. 2021.  

 --------------------------------------------------------------------------------------------------------------------

 (V) - ***Discussion:***

 [8] **Reviewer Concern**: *Emphasize and discuss the added value of the pipeline in obtaining reliable sequences from samples with high ct values. Compare the results obtained using this pipeline with those from other existing pipelines to highlight its superiority.*

**Author Response: **As described in [6], we developed CoVpipe2 as a robust and modular surveillance pipeline focusing on amplification protocols and short-read data. Thus, for samples with high Ct values and where amplification can not yield enough output, CoVpipe2 will mark them as “failed” in the reporting. High Ct samples will usually result in regions (amplicons) with low coverage. Such regions are then automatically masked by “N” bases in the final consensus. We don't think a bioinformatics pipeline should construct any “reliable” consensus genome sequence when the data is insufficient. Thus, it is more important to identify such low-quality samples and flag them with a user warning. Our filtering and reporting aims to fit the needs of large-scale surveillance programs with detailed QC information and provide a quick overview of sample results, to identify such challenging samples easily. Thus, CoVpipe2 helps virologists to identify such problematic samples so that they can be selected for re-sequencing or excluded from downstream analysis. 

 Regarding the comparison to other pipelines, we implicitly did that by selecting the test data sets. Those come from another independent benchmark study (Xiaoli et al. 2022), and we compare our CoVpipe2 results against those from the original benchmark paper. The original authors wrote in their publication:

 > The datasets presented here were generated to help public health laboratories build sequencing and bioinformatics capacity, benchmark different workflows and pipelines, and calibrate QC thresholds to ensure sequencing quality.

 All available pipelines (Tab. 1 in manuscript) excel in various properties. While some strive to have high detection rates for minor variants for research settings, CoVpipe2 was developed to be easily extendable and adjustable to new requirements in surveillance or other viruses [see 9]. Thus, we would like to stick to our decision of utilizing a publicly available and carefully constructed, independent benchmark data set instead of including more samples and pipelines. Our study focuses on presenting the CoVpipe2 implementation and highlighting various implementation decisions in the context of reconstructing robust genome sequences for surveillance tasks. Nevertheless, we agree that another large-scale and up-to-date benchmark study comparing all available pipelines (Tab. 1), including CoVpipe2, would be interesting but is beyond the scope of our Software article. 

 --------------------------------------------------------------------------------------------------------------------

 [9] **Reviewer Concern**: *Provide detailed insights into how this pipeline can be applied to study other viruses*

**Author Response:** The predecessor of CoVpipe2, the snakemake pipeline CoVpipe1, was used to create adapted pipelines for RSV and Influenza. In this context, we discovered that other viruses might need other tools and parameters to reflect their characteristics (genome size, segmentation, reference selection). Also, the needs for final reporting may differ depending on the virus under investigation, and changes in the pipeline may be necessary. Besides, we successfully used CoVpipe2 on Polio and Measles viruses for genome reconstruction and variant calling from short-read sequencing data. In short, for Polio viruses, we sequenced the same 24 samples with Sanger, Illumina, and Nanopore, and CoVpipe2 was able to identify the same variants compared to the other sequencing technologies and associated bioinformatic steps (unpublished preliminary data).  

 In general, the basic software framework - the generic sub-processes of raw data quality control, read alignment, variant calling, and consensus building - and the bioinformatic challenges for data derived from amplicon sequencing are equally applicable to other viruses. CoVpipe2 can serve as a blueprint for other pathogens, especially other unsegmented viruses, and provide first insights into the variants and consensus sequences. Tools, parameters, and thresholds might need careful adjustments depending on the pathogen. Similarly, the downstream analysis might be pathogen-specific, e.g., require pathogen-specific datasets (such as reference sequences for Influenza from Nextclade). We are currently working on a harmonized multi-pathogen pipeline with different profiles (tools, parameter settings) tailored towards specific viruses. Besides, interested users can already run CoVpipe2 on other (non-segmented) viruses by simply switching to another reference genome as we did before successfully for analyzing Polio virus amplicon data (parameter --ref_genome). 

 We extend the “Conclusion” accordingly:

 “We used CoVpipe1 and CoVpipe2, which were initially developed for SARS-CoV-2, to reconstruct the genomes of other viruses. By selecting different reference genomes for polio, measles, RSV, and influenza viruses and modifying the analysis processes for the latter two viruses, we were able to demonstrate the workflow's potential as a universal blueprint for viral genome analysis. This adaptability has been demonstrated by the ability to identify consistent variants and the successful application to different viral characteristics, such as varying genome size and segmentation. However, it should also be noted that for segmented viruses, more customization is required than simply replacing the (non-segmented) reference genome. However, our experience suggests that customization of tools, parameters, and reporting requirements is needed for each virus, pointing to developing a harmonized pipeline for multiple pathogens. CoVpipe2 thus proves to be a robust tool for SARS-CoV-2 and paves the way for broad application in virology research by highlighting its ability to serve as a fundamental framework for building customized pipelines for a wide range of pathogens.”

# Reviewer report: Reviewer response for version 1

**DOI:** 10.5256/f1000research.149827.r203328

**Published:** 2023-09-20

**Authors:** Wolfgang Maier

**Affiliations:** 1 Bioinformatics Group, Department of Computer Science, Albert-Ludwigs-Universitat Freiburg, Freiburg, Baden-Württemberg, Germany

The manuscript by Lataretu et al. describes CoVpipe2, a bioinformatics pipeline for constructing viral consensus sequences from SARS-CoV-2 short sequenced reads obtained using the Illumina platform. It discusses the current state of the pipeline, various design decisions that have led to that state, and typical analysis pitfalls that the authors hope to overcome with their pipeline design.

 The pipeline itself is implemented as a Nextflow pipeline and comes under a free and open-source license, which means that its exact steps and parameters can be explored down to any desired level of detail. Still the authors provide a very helpful overview in the manuscript text and in Figure 1 through both of which the reader can gain a good understanding of the pipeline layout and its components.

 The authors point out the existence of multiple alternative analysis pipelines with similar scope as CoVpipe2 and list many of them in Table 1. I would expect most of these alternative pipelines to produce consensus genomes of similar quality as CoVpipe2, and most of the steps that CoVpipe2 is composed of are relatively standard in the field. From this perspective, the manuscript could be said to lack novelty. Beyond the core steps shared in similar form with many other pipelines, there are, however, some smart extra steps built into CoVpipe2 that are innovative ideas, like screening steps for mixed-infection and recombinant samples and consensus genome annotation with Liftoff.

 More importantly, however, the authors are not just advertising yet another pipeline for SARS-CoV-2 genome analysis, but their manuscript is the kind of documentation that you wish every such pipeline came with: it explains not only individual analysis steps, but also their purpose, special analysis tweaks found to be necessary, and provides links to all relevant resources.

 In summary, the manuscript describes a robust and mature resource for reproducible data analysis and does an excellent job at that. I enjoyed reading it and even though I've spent a considerable amount of time on developing similar pipelines I still picked up a few new ideas from it.

 I have no concerns regarding publication of this valuable manuscript, just one comment that the authors may wish to address in the manuscript directly or in a separate reply:

 For some steps in CoVpipe2 it seems there would have been several tools to choose from, and I'm wondering whether the authors of the pipeline have evaluated alternatives. In particular, I'd be interested in learning why freebayes was chosen as the variant caller and why primer trimming is done with BAMclipper, as I don't think these two tools are used by many other comparable pipelines. If the authors had specific reasons to prefer these tools over alternatives, it might add to the value of the manuscript if these were added to the text.

 Beyond that, I have found a small number of inconsistencies and typos that I think should be fixed before publication:

 1. the authors have done a very careful citation job, in general, but I think the following resources/specifications also deserve links/citations:

- Zenodo
- The "precalculated Kraken2 database" deposited at Zenodo
- The BEDPE format ( https://bedtools.readthedocs.io/en/latest/content/general-usage.html#bedpe-format)
- Instead of providing a general anaconda.org link (as done twice in the Methods section), it would be more helpful to provide direct links to the latest versions of pangolin and nextclade ( https://anaconda.org/bioconda/pangolin and https://anaconda.org/bioconda/nextclade), which also includes channel information.

2. In the introduction " *While sequencing intensity and turnaround times on variant detection increased in different countries*" is probably intended to mean increasing sequencing intensity, but *decreasing* turnaround times?

 3. In the introduction, when DESH genomes statistics are given, the numbers should be 1.2 million and 1.1 million (period instead of comma), and in the discussion of Dataset 5 -> Lineages " *for each CoVpip2-GISAD pair*" has a typo in the pipeline name.

Are the conclusions about the tool and its performance adequately supported by the findings presented in the article?

Yes

Is the rationale for developing the new software tool clearly explained?

Yes

Is the description of the software tool technically sound?

Yes

Are sufficient details of the code, methods and analysis (if applicable) provided to allow replication of the software development and its use by others?

Yes

Is sufficient information provided to allow interpretation of the expected output datasets and any results generated using the tool?

Yes

Reviewer Expertise:

Bioinformatics, pathogen genomics, genetics

I confirm that I have read this submission and believe that I have an appropriate level of expertise to confirm that it is of an acceptable scientific standard.

# Response

**Published:** 2024-04-10

**Authors:** Martin Hölzer

**Affiliations:** Genome Competence Center (MF1), Robert Koch Institute, Berlin, Berlin, Germany

[1] 

 Reviewer Question: *For some steps in CoVpipe2 it seems there would have been several tools to choose from, and I'm wondering whether the authors of the pipeline have evaluated alternatives. In particular, I'd be interested in learning why freebayes was chosen as the variant caller and why primer trimming is done with BAMclipper, as I don't think these two tools are used by many other comparable pipelines. If the authors had specific reasons to prefer these tools over alternatives, it might add to the value of the manuscript if these were added to the text.*

 Author Response: Thanks for the question. You are absolutely right; as with almost any bioinformatics pipeline, there are different options when choosing tools for steps such as quality control, mapping, and variant calling. Our final selection of tools is based on our experience in analyzing sequencing data throughout the SARS-CoV-2 pandemic. Especially in the early days, we performed various internal benchmarks on SARS-CoV-2 Illumina data and manually investigated mapping results and variant calls together with colleagues from our expert unit for respiratory viruses. 

 Thus, our main objective in CoVpipe2 was to reliably detect variants with high allelic depth and good read support. Low-frequency variants were not the primary focus of the pipeline, as the tool is intended to reconstruct robust consensus genomes from patient samples that can be used for genomic surveillance. However, if a user wants to use CoVpipe2 for different research questions, the implementation allows full customization of the necessary parameters (allele frequency, genotype adjustment, …) By screening the literature and examining other pipelines and community standards, we carefully selected the tools that performed best in our internal benchmarks for SARS-CoV-2 short-read data.  

 Regarding variant calling, we first tested LoFreq (Wilm *et al.* 2012). Although very sensitive, LoFreq lacks a strong genotyping module that was crucial for our downstream processing of the called variants. Furthermore, the output files were hard to process (non-standard VCF formats). We implemented GATK as a second choice, which is a standard tool for eukaryotic genomic variant calling (McKenna *et al*. (2010), Van der Auwera & O'Connor (2020)) but was also shown to perform well on non-human targets (Lefouili *et al.* 2022). Performance and output standards were excellent, but it turned out that GATK misses a low amount of viral genomic variants in some samples, although multisample calling was employed. Single false negative variants, which we identified via a comprehensive investigation of the BAM files, were deemed to be too important to stick with the tool. Finally, we chose freebayes (Garrison *et al*. 2012), which excelled with high performance, high precision, and output files that were straightforward to process in downstream steps of the pipeline.

 In addition to the variant callers, there is indeed a large selection of quality processing tools. We opted for fastp rather than Trimmomatic because the processing speed is much faster, and the output quality is at least as good. It was shown, that “data filtered by Trimmomatic, SOAPNuke, Cutadapt and fastp were detected with 7174, 7040, 6942 and 6708 false positive variants respectively” (Chen *et al*. 2018), highlighting that fastp preprocessing can even improve the specificity of downstream analysis. In addition, fastp is equipped with a broader portfolio of parameter options.  

 Another crucial step in read processing is primer clipping, which may promote mapping artifacts and dilution of variant calls if done before read alignment. If InDels occur close to the end of amplicons, a gap open penalty is more expensive than a few mismatches in mapping. For example, this caused trouble with the Spike DEL69/70 in several amplicon kits and made it necessary to use the artificial primer as a mapping anchor. Hence we replaced cutadapt, which clips adapters before mapping, with BAMclipper, which removes adapters after mapping. In our opinion, primer clipping should generally be performed after mapping in reference-based analysis. We, therefore, also discuss late primer clipping more prominently in connection with CoVpipe2. 

 Furthermore, we fully agree that continuous benchmarking is a necessary process to adapt pipelines to changing wet lab procedures, adapted priming schemes, and pathogen evolution. If we find problems, we will also adapt CoVpipe2 accordingly and release new stable versions for reproducible research.  

 We added the following text to the manuscript:

 “We carefully selected the bioinformatics tools integrated into CoVpipe2 based on internal benchmarks and in-depth manual reviews of sequencing data, mapping results, and called variants. Based on our hands-on experience with SARS-CoV-2 sequencing datasets during the pandemic, this approach ensured that tools that can robustly detect high allele depth and well-covered variants are used for detection. Despite the primary goal of CoVpipe2 to identify high-confidence variants to reconstruct robust consensus genomes for genomic surveillance, the pipeline also provides flexibility for adaptation to different research environments.”

 --------------------------------------------------------------------------------------------------------------------------

 [2]

 Reviewer Question: *the authors have done a very careful citation job, in general, but I think the following resources/specifications also deserve links/citations]:*

 Author Response: Thanks for the comment. We fully agree and added more precise references for various sources such as Zenodo, the custom Kraken 2 database, BEDPE format, and Nextcalde/pangolin on anaconda.org.

 --------------------------------------------------------------------------------------------------------------------------

 [3]

 Reviewer Question: In the introduction "While sequencing intensity and turnaround times on variant detection increased in different countries" is probably intended to mean increasing sequencing intensity, but *decreasing* turnaround times?

 Author Response: Yes, you are right; thanks for catching this. We changed the text accordingly: 

 “While sequencing intensity increased and turnaround times on variant detection decreased in different countries, there are also major disparities between high-, low- and middle-income countries in the SARS-CoV-2 global genomic surveillance efforts.”

 --------------------------------------------------------------------------------------------------------------------------

 [4]

 Reviewer Question: *In the introduction, when DESH genomes statistics are given, the numbers should be 1.2 million and 1.1 million (period instead of comma), and in the discussion of Dataset 5 -> Lineages "for each CoVpip2-GISAD pair" has a typo in the pipeline name.*

 Author Response: Thanks, we corrected that.
